# Travel beyond Clinical Uses of Fiber Reinforced Composites (FRCs) in Dentistry: A Review of Past Employments, Present Applications, and Future Perspectives

**DOI:** 10.1155/2018/1498901

**Published:** 2018-10-22

**Authors:** Andrea Scribante, Pekka K. Vallittu, Mutlu Özcan, Lippo V. J. Lassila, Paola Gandini, Maria Francesca Sfondrini

**Affiliations:** ^1^Unit of Orthodontics and Paediatric Dentistry, Section of Dentistry, Department of Clinical, Surgical, Diagnostic and Paediatric Sciences, University of Pavia, Italy; ^2^Department of Biomaterial Science and Turku Clinical Biomaterials Centre (TCBC), Institute of Dentistry, University of Turku, Turku, Finland; ^3^City of Turku, Welfare Division, Turku, Finland; ^4^University of Zurich, Center for Dental and Oral Medicine, Dental Materials Unit, Clinic for Fixed and Removable Prosthodontics and Dental Materials Science, Zurich, Switzerland

## Abstract

The reinforcement of resins with short or long fibers has multiple applications in various engineering and biomedical fields. The use of fiber reinforced composites (FRCs) in dentistry has been described in the literature from more than 40 years. In vitro studies evaluated mechanical properties such as flexural strength, fatigue resistance, fracture strength, layer thickness, bacterial adhesion, bonding characteristics with long fibers, woven fibers, and FRC posts. Also, multiple clinical applications such as replacement of missing teeth by resin-bonded adhesive fixed dental prostheses of various kinds, reinforcement elements of dentures or pontics, and direct construction of posts and cores have been investigated. In orthodontics, FRCs have been used also for active and passive orthodontic applications, such as anchorage units, en-masse movement units, and postorthodontic tooth retention. FRCs have been extensively tested in the literature, but today the advances in new technologies involving the introduction of nanofillers or new fibers along with understanding the design principles of FRC devices open new fields of research for these materials both in vitro and in vivo. The present review describes past and present applications of FRCs and introduces some future perspectives on the use of these materials.

## 1. Introduction

Fiber-reinforced composites (FRCs) have been studied for biomedical applications for over 40 years [1] and were specifically developed in dental field over 25 years ago [2]. FRCs are composite materials with three different components: the matrix (continuous phase), the fibers (dispersed phase), and the interphase region (interphase). In general, the matrix phase is composed of polymerizable monomers that convert from a fluid to a highly crosslinked polymer upon exposure to visible light. Alternatively, linear polymers such as poly(methyl methacrylate) can be utilized in thermoplasticization process or in monomeric form [3, 4]. With cross-linkable resin systems, the light exposure catalyzes the formation of radicals that induce polymerization. The fibers are added primarily because of high stiffness/weight (specific modulus) and strength/weight (specific strength) when compared with other structural materials [5]. Essentially, fibers act as the reinforcing phases when a load is applied to the composite.

The incorporation of fibers into the organic matrix provides material-specific characteristics. Fiber bundles can be discontinuous or continuous, with randomly directed or directional fibers. The strongest FRC devices are typically made of continuous unidirectional fibers [6]. Fibers can be made of different materials, such as carbon, aramid, polyethylene, or glass. Glass fibers vary according to their composition and are the commonly used fibers in dentistry [7]. This is due to their transparency and beneficial surface chemistry, which allows their adhesion to resin [8]. In fact, adhesion of FRC frameworks has been reported to be reliable for long bundles [9], short bundles [10], and nets [11]. The adhesion of fibers is primarily based on the presence of hydroxyl groups on the surface of glass fibers and the reaction of the groups with resin monomers via silane coupling agents [12, 13].

Some FRCs are hand fabricated, with a polymeric matrix added to the fibers at chairside. This approach might not produce an effective composite, because coupling between the fiber and the polymer might be inadequate and leave voids. On the other hand, partial- or full- preimpregnated FRCs are partially or fully polymerized continuous long fibers, which offer superior properties, because they combine both polymer and fibers [14].

Reinforcement of polymers with long, continuous fibers is an effective mean for engineering materials for many applications. FRCs have been proposed in many fields in dentistry for different purposes, namely, prosthodontics, endodontics, conservative dentistry, orthodontics, periodontology, and paediatric dentistry (Table 1). Previous studies reported FRCs used for veneered fixed dental elements [15], root canal posts [16], filling resin composites [17], periodontal splints [18], orthodontic retainers [19, 20], and orthodontic brackets [21]. In addition, temporary fixed dental prostheses (FDP) [22], reinforcement of removable devices, [23] and repairs of conventional restorations [24] have been reported. Finally, also oral and maxillofacial surgery purposes have been described, as FRCs can be used for implants and bone substitutes for craniofacial bone reconstruction [25].

## 2. Literature Review and Brief Bibliometric Report

A broad search on Scopus Database has been conducted using the following MeSH terms:

  TITLE-ABS-KEY ( fiber AND reinforced AND composite )

The search strategy included an initial analysis of the results in the specific Scopus sections dedicated to the different document types, thus allowing to highlight the kind of document (articles; conference papers; reviews; book chapters; articles in press; book chapters; editorial; erratum; note; and conference review). No exclusion criteria have been applied in order to provide a whole publications count.

Furthermore, the analysis has been refined with the function “search within results,” with the following MeSH terms for each discipline considered in the investigation:

  ( TITLE-ABS-KEY ( fiber AND reinforced AND composite ) ) AND ( dental AND materials )
  ( TITLE-ABS-KEY ( fiber AND reinforced AND composite ) ) AND ( prosthodontics )
  ( TITLE-ABS-KEY ( fiber AND reinforced AND composite ) ) AND ( endodontics )
  ( TITLE-ABS-KEY ( fiber AND reinforced AND composite ) ) AND ( conservative AND dentistry )
  ( TITLE-ABS-KEY ( fiber AND reinforced AND composite ) ) AND ( orthodontics )
  ( TITLE-ABS-KEY ( fiber AND reinforced AND composite ) ) AND ( periodontology )
  ( TITLE-ABS-KEY ( fiber AND reinforced AND composite ) ) AND ( paediatric AND dentistry )

**Figure 1:**
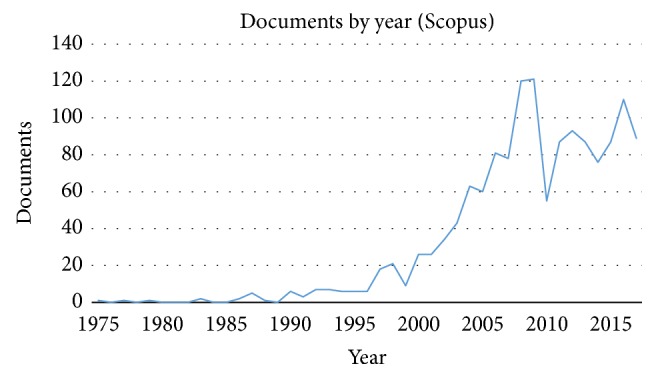
Number of research papers published on fiber reinforced composites by year in the field of dentistry (source: Scopus database).

The results of this research revealed that, today in the literature, more than 80.000 documents have been published on FRC materials when Scopus-indexed journals are considered. Based on the published material, it could be stated that the main subjects of investigation were engineering, materials science, physics, and chemistry. In total, 1797 studies have been reported in medical field, of which 1473 were on dental related topics. This remarkable production mainly consists of original articles (1333). Other contributions are conference papers (62), reviews (45), and book chapters (19). The main part of this type of research (1444 documents) was published in sources that require university/hospitals special access or consultation under payment, whereas only 29 documents were free access with an open access route. The research on FRC materials in dentistry seemed to start in 1975 [1] although first reports were already from the 1960s [26, 27]. However, until 1989, only 13 reports have been published on the FRCs (Figure 1). After 1990, the FRC topic started gaining increasing popularity in dental research. Starting from 2004 over 50 documents have been published each year until today, with the highest number of 121 reports in 2009. This is followed by 110 published documents in 2016 and 89 in 2017. During the first 4 months of 2018, already 35 Scopus-indexed manuscripts about the FRC in dentistry have been published, thus confirming that the interest in the FRC topic is still very high. In fact, new technologies allow continuous improvement of materials and techniques, opening new investigation and application fields of FRCs.

Among various dental fields, the main topic of published material was on material properties (1294) where 897 documents were on prosthodontics, 448 on endodontics, 215 on conservative dentistry, 194 on orthodontics, 164 on periodontology, and 132 on paediatric dentistry (Table 2). Many studies have a multidisciplinary approach and present cross-matter subjects. While most of the published research was in vitro, clinical trials were limited to 70 documents.

## 3. Clinical Applications in Prosthodontics

The main application of FRCs in dentistry is related to provisional or definitive prosthodontics. By using FRCs, FDPs and veneers can be realized in a minimal invasive fashion, utilizing combinations of various kinds of adhering and retentive elements [22]. A resin bonded FRC prosthesis may contain inlays/onlays, surface bonding wings, and crowns. Direct and indirect frameworks can be made also immediately after extraction of tooth [Cramer et al., 2011].

FRC FDPs could be fabricated as surface-retained, inlay-retained, or full coverage crown retained prostheses [28]. The fabrication could be realized directly in the mouth or can include prefabricated pontics, simplifying the fabrication technique and providing more predictable outcomes.

The results of mechanical [29] and adhesion [30] properties of FRC frameworks appear to be encouraging. In addition, FRCs can be used in the repair of existing conventional prosthetic devices. Repairs of veneers of porcelain-fused-to-metal restorations with resin composite veneers can be made using woven glass fiber reinforcement, thus increasing the strength of the repair [31, 32]. In addition, removable devices could be reinforced using FRCs [23]. Finally, FRCs can be used in indirect pontic fabrication, also in combination with CAD/CAM based technologies [33–35].

## 4. Clinical Applications in Conservative Dentistry

The applications of FRCs in conservative dentistry mainly consist of direct composite restorations. The advantages of the use of FRCs over conventional filling materials are related to their biomimetic properties. In fact, the dental restorations ideally would be as minimally invasive as possible and substitute the missing hard dental tissues resembling mechanical features and properties of natural teeth [36]. Following this principle, a bilayered approach in dental restorations has been proposed in which lost dentin is replaced by though short FRCs and enamel by surface layer of particulate filler composite resin. Several authors have shown that the FRC substructure supports the composite restoration and serves as a crack-prevention layer [37]. In fact, FRCs have been reported to have superior physical properties and fracture toughness compared to unreinforced composites [38]. In addition, polymerization shrinkage and depth of cure of FRCs have been reported to be superior to conventional resin composites [36].

Superior mechanical properties of FRCs could improve their bond durability with universal adhesives, even if there is little evidence comparing the bond durability of FRC to dentin with that of other composite resins [10]. On the other hand, bilayered biomimetic technique is recommended for direct coronal restorations of teeth with large cavities in high stress-bearing areas [24, 39, 40].

## 5. Clinical Applications in Endodontics

In endodontic clinical practice, the use of FRCs is mainly reported as root canal anchoring system. Studies evaluated both prefabricated and individualized FRC posts [16, 41–43]. Root canal walls restored with individually formed FRC posts displayed higher fracture resistance than those restored with only resin composite [44–46]. Bond strength to flared root canal dentin is promising also for FRC posts both used in combination with self-adhesive and glass ionomer cements and FRCs achieved better performances, even in combination with bulk fill resin composite [47]. However, after aging, mechanical behavior of posts significantly decreased when compared with values at baseline [48]. In addition, special attention should be paid to the bonding of luting cement and core-built-up composite to FRC post itself: only FRC post with interpenetrating polymer network containing polymer matrix can provide reliable bonding to resin luting cements and resin based materials in general [42, 49, 50].

Generally, FRCs present limited radio-opacity due to the low concentration of radio-opaque elements. This shortcoming of E-glass fiber would limit its application in dentistry as sufficient radio opacity is highly desirable for dental materials. The addition of synthesized iodine containing a new methacrylate monomer HMTIB has been tested to increase the radio opacity of FRCs with the results showing that FRCs present higher radiopacity than natural tooth enamel [51].

Finally, in the field of endodontics, FRCs showed excellent integration with other new technologies such as laser applications [52] and CAD/CAM [53, 54].

## 6. Clinical Applications in Orthodontics

The main use of FRCs in clinical orthodontics is as fixed retention [14]. After orthodontic treatment, the need for maintaining the teeth in correct position is crucial for long term stability of clinical results. These bonded retainers appear to be both relatively independent of patient cooperation and well accepted by patients [55]. Bond strength is reported to be sufficient both on enamel [56] and on dentin [10]. Clinical reliability is also reported to be successful for moderate time [57].

A great advantage of FRC splints over conventional metallic retention is aesthetics. Fibers are barely invisible and do not affect the translucency of teeth [Karaman et al., 2002]. This aspect is important, considering the higher number of adult patients who request an orthodontic therapy. Finally, FRCs are metal-free and are indicated for adult and young patients screened by Nuclear Magnetic Resonance or in subjects allergic to metals. On the other hand, FRC splints are more rigid than conventional metallic splints, thus leading to a higher ankyloses risk of teeth involved. However, the application of FRC with a spot-bonding technique has been proposed, in order to reduce framework rigidity, thus allowing physiologic tooth movement [58].

Clinical success of FRC resins has been reported also for space maintainer purpose [59]. The early loss of deciduous molars is a frequently encountered problem in dentistry and, if untreated, it could evolve in various orthodontic problems. Space maintainers are developed to prevent the loss of the space. FRC space maintainers can be prepared on plaster models of patients and fixed directly to the adjacent teeth [60].

In addition to stabilization uses, in orthodontics, FRCs have been proposed also for active tooth movement. Groups of two or more teeth can be splinted with FRCs and moved “en masse” with sectional mechanics [61].

One other application of FRCs has been proposed as innovative materials for fabrication of brackets [21] and wires [62]; yet only a few research papers have been conducted on the topic.

## 7. Clinical Applications in Periodontology

Periodontal or posttraumatic FRC splints have been reported in clinical periodontology. Splints are used to stabilize teeth, which have become loose as a result of supporting bone loss as a consequence of periodontal disease. The main advantage of stabilization splints is the reduction of tooth mobility. [18]. FRC periodontal or posttraumatic splints have been reported to have reliable long term stability [63]. In fact, fiber reinforced frameworks showed higher flexural forces when compared with conventional metallic wires [64]. Moreover, FRC splints showed high flexural resistance also when polymerized directly with polymerization lamp without laboratory oven postpolymerization, thus reducing the number of clinical steps and number of appointments for the patients [65]. The common failure types are debonding and fractures. In fact, the splinting with FRC materials of periodontally compromised teeth that have different mobility grade is prone to debonding, with the mobility grade as main causative factor. However, FRC splints can be easily repaired, so in many cases it is not necessary to completely debond the framework with the substitution with a new one [66].

## 8. Clinical Applications in Paediatric Dentistry

In paediatric dentistry FRCs can be used in almost all the fields as described above: restorations, space maintainers, splints, or other frameworks [67]. The main difference is that the enamel of primary teeth is significantly different compared to permanent enamel. The differences have been mainly detected in composition [68], mechanical characteristics [69], bond strength [70], and clinical performance [71]. However, the FRC devices used in paediatric dentistry showed acceptable clinical performance [71], durability [72], and ease of use [73].

## 9. Clinical Applications in Oral and Maxillofacial Surgery

The use of FRCs has been recently reported also in oral and maxillofacial surgery. These materials can be applied in oral implantology for bone replacing and bone anchoring implants. The rationale for this application is that, although metal implants have successfully been used for decades, devices made out of metals do not meet all clinical requirements. Metal objects may interfere with some medical imaging systems, while their stiffness also differs from natural bone and may cause stress shielding and overloading of bone. Glass fibers are responsible for the load-bearing capacity of the implant, while the dissolution of bioactive glass particles supports bone bonding and provides antimicrobial properties for the implant [74].

Moreover, FRCs materials can be used in maxillofacial discipline for orbital floor implants [75], cranioplasty implants [25], and craniofacial bone reconstruction [76].

## 10. Advantages of the Use of FRCs

The main advantages of the use of FRCs over conventional materials are mainly due to their easy manipulation and high mechanical properties especially in dynamic loading conditions. For many FRC applications, no or minimal laboratory work is needed and often frameworks can be prepared at chairside, directly in the oral cavity [77]. The other positive characteristic is the high aesthetics achieved with these materials over metal reinforced alternatives [8]. Finally, the absence of metallic parts in the FRC structure allows their use also in patients allergic to nickel or other metals. Noteworthy is that FRCs can be indicated in patients who need to undergo nuclear magnetic resonance exams [78].

## 11. Limitations of the Use of FRCs

The main limitations of FRC clinical use are that, even though many in vitro studies have been conducted, research is still lacking regarding long-term clinical performance. The most important weakness of FRC is the interface between the fiber and the organic matrix. Intraoral hydrolysis and degradation weaken this interface and failure can occur. Maybe this might also be a reason for missing long-term results.

Principal failure reasons of FRC devices are fracture and delamination but such events could be easily repaired with resin composite materials [66].

Finally, the higher cost than unreinforced or metallic materials is a factor that has to be considered for a global evaluation of FRC employment.

## 12. New Features and Future Applications

Future research on FRCs needs to focus on many aspects such as optimization of the design of the frameworks in FRC devices [79], incorporation of bioactive minerals into the reinforced resin composites, and the change to fiber binding matrix from resin base to inorganic type [80].

Another improvement is related to nanotechnology, with the production of functional structures in the range of 0.1-100 nm by various physical or chemical methods. Dental nanocomposites provided a cosmetically acceptable result with excellent mechanical properties [19, 20]. The main point involved with this new trend is the addition of nanofillers particles to resin-based dental materials [81]. The utilization of continuous [82] and discontinuous [83] nanofillers has been proposed in conjunction with FRCs.

FRC utilization has been proposed also in combination with Computer-Aided-Design/Computer-Aided-Machining (CAD/CAM) technologies. The interaction between the two technologies seems to be promising based on limited information [35].

One other field where FRCs are starting to be utilized is implantology. Implant applications could benefit from certain biomechanical properties of FRCs, and the possibility of incorporating additional bioactive components into the implant structure may open new research fields [74].

FRCs have been suggested for tissue engineering for orthopaedic scaffolds [80]. As biocompatibility results are promising, FRC biomaterials developed may constitute an optimized alternative to the other materials used for the reconstruction of craniofacial bone defects [76].

The research options with FRC materials are open and future reports about the topic are expected to widen FRC utilization in both dental and medical fields.

## Figures and Tables

**Table 1:** Main clinical applications of fiber reinforced composites in dentistry.

**Dentistry field**	**Clinical use**
Prosthodontics	Provisional or definitive fixed dental prostheses, veneers, direct or indirect pontics, and repair of removable devices
Endodontics	Prefabricated or customized root canal anchoring systems
Conservative dentistry	Direct and indirect fillings, inlays, and overlays
Orthodontics	Retention splints, space maintainers, active “en-masse” units, metal-free brackets, and orthodontic wires
Periodontology	Periodontal splints and posttraumatic splints
Paediatric dentistry	Crowns in primary molars, splints, space maintainers, and direct fillings

**Table 2:** Number of studies published on FRCs in various dental fields such as prosthodontics, endodontics, conservative dentistry, orthodontics, periodontology, and paediatric dentistry. Note that the majority of the studies are multidisciplinary and present cross-matter subjects.

**Document type**	**Number of studies**	**Materials properties**	**Prosthod**	**Endod**	**Conserv dent**	**Orthod**	**Periodontol**	**Paediatr dent**
Articles	1333	1186	841	432	200	182	147	116
Conference papers	62	46	15	4	2	5		2
Reviews	45	40	23	8	6	6	9	7
Book chapters	19	15	10	1	5		4	6
Articles in press	4	2	4	2	1		2	1
Book chapters	3	1				1		
Editorial	2	2	2	1	1			
Erratum	2	1					1	
Note	2		2					
Conference review	1	1					1	

**Total**	**1473**	**1294**	**897**	**448**	**215**	**194**	**164**	**132**

## Data Availability

Data are available upon request at andrea.scribante@unipv.it.
